# Association of Soluble HLA-G with Acute Rejection Episodes and Early Development of Bronchiolitis Obliterans in Lung Transplantation

**DOI:** 10.1371/journal.pone.0103643

**Published:** 2014-07-28

**Authors:** Steven R. White, Timothy Floreth, Chuanhong Liao, Sangeeta M. Bhorade

**Affiliations:** 1 Departments of Medicine and Health Studies, University of Chicago, Chicago, Illinois, United States of America; 2 Department of Medicine, Northwestern University, Chicago, Illinois, United States of America; Cedars-Sinai Medical Center, United States of America

## Abstract

Lung transplantation has evolved into a life-saving therapy for select patients with end-stage lung diseases. However, long-term survival remains limited because of bronchiolitis obliterans syndrome (BOS). Soluble HLA-G, a mediator of adaptive immunity that modulates regulatory T cells and certain classes of effector T cells, may be a useful marker of survival free of BOS. We conducted a retrospective, single-center, pilot review of 38 lung transplant recipients who underwent collection of serum and bronchoalveolar lavage fluid 3, 6 and 12 months after transplantation, and compared soluble HLA-G concentrations in each to the presence of type A rejection and lymphocytic bronchiolitis in the first 12 months and to the presence of BOS at 24 months after transplantation. Lung soluble HLA-G concentrations were directly related to the presence of type A rejection but not to lymphocytic bronchiolitis. Our data demonstrate that soluble HLA-G concentrations in bronchoalveolar lavage but not in serum correlates with the number of acute rejection episodes in the first 12 months after lung transplantation, and thus may be a reactive marker of rejection.

## Introduction

Lung transplantation (LT) remains the best hope for selected patients with end-stage lung diseases. Chronic allograft rejection, clinically manifested as bronchiolitis obliterans syndrome (BOS), remains a major limitation to long-term survival: BOS occurs in 40–60% of lung transplant recipients within 4 years and is the leading cause of death after the first year, despite advances in the use of immunosuppressive therapy [1]. Although several alloimmune-dependent and independent events have been considered as risk factors for BOS, the most common and consistently identified factor associated with the development of BOS is acute lung allograft rejection episodes and alloimmune T-cell reactivity [2], [3].

HLA-G is a major histocompatibility complex class I antigen encoded by a gene on chromosome 6p21 [4]. Two HLA-G isoforms exist outside the placenta: membrane-bound G1 and soluble G5 (sHLA-G) that due to alternative splicing lacks the transmembrane and intracellular domains of G1 [5]. HLA-G binds the inhibitory receptor Ig-like transcript (ILT)2/LILRB1/CD85j, expressed by human NK cells, monocytes, T cells, B cells and dendritic cells [6], and the myeloid-specific ILT4/LILRB2/CD85d receptor [7]. HLA-G has effects on both CD4+FoxP3+ regulatory T (Treg) cells and on alloreactive recipient alloreactive CD4+ and CD8+ effector T (Teff) cells that may be beneficial in transplantation: it induces expansion of Treg cells [8], inhibits both NK cell- and CD8+ T cell-mediated cytolysis [9], suppresses CD4+ T cell alloproliferative responses [10], and induces apoptosis of CD8+ T cells [11]. Perhaps more important for long-term tolerance, HLA-G-bearing antigen-presenting cells also induce the differentiation of CD4+ T cells into suppressor cells [12], [13].

HLA-G has been demonstrated in heart transplant allografts: patients with higher HLA-G expression had fewer acute rejection episodes (AREs) and less evidence for chronic rejection [14], [15]. Circulating sHLA-G was seen only in patients with HLA-G expression in the heart allograft, suggesting the allograft as the source [14]. Similar results were seen in patients following liver [16] and renal [17] transplantation. Suppressor T cells were present in increased number in liver and liver-kidney transplant patients who express HLA-G at high levels [18]. In one recent single-center, retrospective study of 64 LT recipients within the first year of transplant, HLA-G expression was seen in both bronchial and alveolar epithelial cells most frequently in stable patients but less so in patients with frequent AREs or in patients with BOS [19]. This study did not evaluate the presence of either circulating or local (lung) sHLA-G, however.

We have previously demonstrated that low numbers of FoxP3+ Treg cells are associated with accelerated rejection and the development of BOS in LT [20]. Other investigators have demonstrated that increasing the number/function of Treg cells is associated with less alloreactivity in GVHD [21]. Given the association between the presence of HLA-G and other solid-organ transplantation and the potential modulatory role of HLA-G on Treg and Teff function, we asked whether there was an association between HLA-G locally in the recipient lung in the first year after LT and subsequent BOS. To answer this question, we examined a respective cohort of LT recipients to compare the presence of sHLA-G in plasma and in bronchoalveolar lavage (BAL) fluid collected in the first year to the number of acute rejection episodes in the first year and the appearance of BOS after transplantation.

## Methods

### Ethics statement

Approval was obtained from the University of Chicago Institutional Review Board (IRB) for this study, and was continuously updated as required during the study. Informed written consent done on forms approved by the IRB was obtained from all patients included in this analysis prior to their participation.

### Patient population

Adult subjects receiving a single or bilateral sequential lung transplant from June, 2006 to September, 2011 at the University of Chicago Hospitals were evaluated. Clinical data, blood samples, and BAL fluid collected by bronchoscopy in the first 12 months after transplantation, and clinical status and pathology samples to determine the presence or absence of acute rejection episodes and BOS in the first 48 months after transplantation, were evaluated. As the point of the study was to evaluate a potential marker for the development of BOS, patients had to survive for 12 months or longer after transplantation to be included in this study.

### Immunosuppression

Baseline immunosuppression for all patients included tacrolimus (target trough level: 10 ng/mL), azathioprine (2 mg/kg/day), and prednisone (tapered to 5 mg/day by 3 months post-transplantation). Daclizumab induction therapy was administered to all patients per the manufacturer's instructions. Immunosuppression was changed because of declining pulmonary function per the discretion of the attending transplant physician.

### Bronchoscopy samples

We have previously described these methods [20]. Specimens were collected during surveillance bronchoscopies in the first 12 months post-transplantation. For BAL, one 60 mL and one 30 mL aliquot were instilled into the distal airways and aspirated. In general, 40 to 50 mL of BAL fluid was recovered. An aliquot of this recovered fluid was processed by clinical laboratories to assess clinical infection. Fluid to be used for mediator analysis was centrifuged at 300× *g* and 4°C for 10 min, after which the supernatant was removed, passed through a 1.2-µm filter, and frozen at −80°C until used. Cell pellets were also frozen at −80°C until analyzed. For transbronchial biopsies, samples were collected by standard technique from the recipient lung and processed for evaluation of rejection.

### Plasma samples

Blood was collected on the same day as bronchoscopy in heparin-containing tubes and immediately placed on ice. Plasma was separated by centrifugation and stored in aliquots at −80°C until use.

### Acute rejection and BOS

Acute rejection was determined by histological analysis of transbronchial biopsies obtained during each surveillance bronchoscopy and clinical bronchoscopies. Acute rejection was graded in accordance with International Society of Heart and Lung Transplantation (ISHLT) guidelines [22], [23]. All analyses included episodes of both grade A rejection (RA) and lymphocytic bronchiolitis (LB). All rejection episodes that met criteria were included in the data analysis. Determination of BOS was done periodically at clinical encounters for each subject using standard spirometry definitions.

### Measurement of sHLA-G

sHLA-G was measured in plasma and in BAL fluid using ELISA (Exbio, Inc., Czech Republic). The capture antibody, MEM-G/9, recognizes both shed G1 and soluble G5. The limit of sensitivity is ∼1 U/ml. Concentrations in BAL fluid were not normalized for BAL fluid protein content or other markers.

### Data analysis

Clinical data are expressed as the mean ± standard deviation or as the median with interquartile ranges. When HLA-G concentrations were below the limit of detection the value was recorded as ‘0’. Results were compared using the non-parametric Kruskal-Wallis test. The Spearman correlation was used to determine associations between HLA-G concentrations and grade A or B rejection. The associations between HLA-G, RA or LB and mortality or BOS-free survival were analyzed using Kaplan–Meier analysis and Cox regression model. The log-rank test was used to compare differences of groups. The survival time was measured from the beginning of lung transplant to the date of death or to the end of the study (Jan. 25, 2013) and BOS-free survival was calculated from lung transplant date to the first of observation of BOS or death or the end of study, whichever was earlier. A p value less than 0.05 was considered significant. The data was analyzed using the statistic software Stata/SE 13.0 and IBM SPSS Statistics 20.

## Results

### Demographics and survival

**Figure 1:**
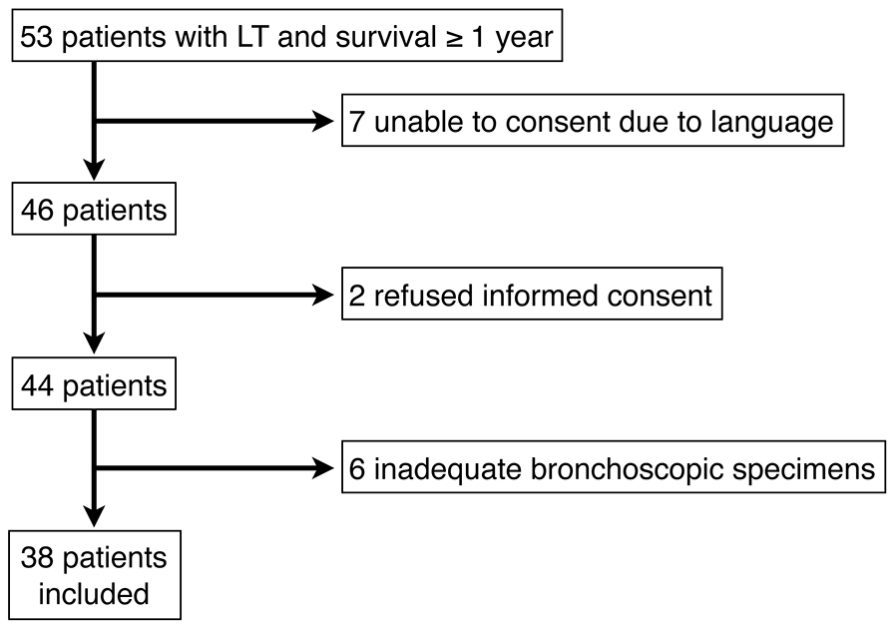
Study enrollment.

We performed 53 lung transplants in the time period of this study in which patients survived for 1 year or longer; of these 38 subjects were eligible for inclusion (Figure 1). Subjects characteristics are shown in Table 1. Of the 38 subjects, 28 survived the length of time recorded in the study (mean survival 4.12±1.73 years), whereas 10 subjects died after lung transplant (mean survival 2.77±1.81 years). These subjects were included in our data analysis. BOS-free survival was 3.22±1.49 years in the 15 subjects recorded as not having a clinical diagnosis of BOS during the study, and 1.07±1.62 years in the 23 subjects who did develop clinically-diagnosed BOS. There were no differences in overall survival based on gender, but median BOS-free survival was greater in male subjects: 3.84 years (1.94 to 5.75 years by 95% confidence interval) versus 1.73 years (1.03 to 2.43 years by 95% confidence interval) for female subjects. There were no significant differences in survival or BOS-free survival based on race, type of transplant (single versus bilateral sequential) or diagnosis at the time of transplantation.

**Table 1:** Demographic characteristics of 38 subjects in study.

Age at time of transplantation, years (SD)	
	58.2 (12.3)
Gender, N (%)	
Female	10 (26.3)
Male	28 (73.7)
Type of transplant*, N (%)	
Single lung	24 (64.9)
Bilateral sequential lung	13 (35.1)
Race, N (%)	
European ancestry	24 (63.2)
African ancestry	4 (10.5)
Hispanic ancestry	4 (10.5)
Other	6 (15.7)
Diagnosis at time of transplantation, N (%)	
IPF	20 (52.6)
COPD	12 (31.6)
CF	3 (7.9)
Other	3 (7.9)

*1 missing.

### Rejection

**Figure 2:**
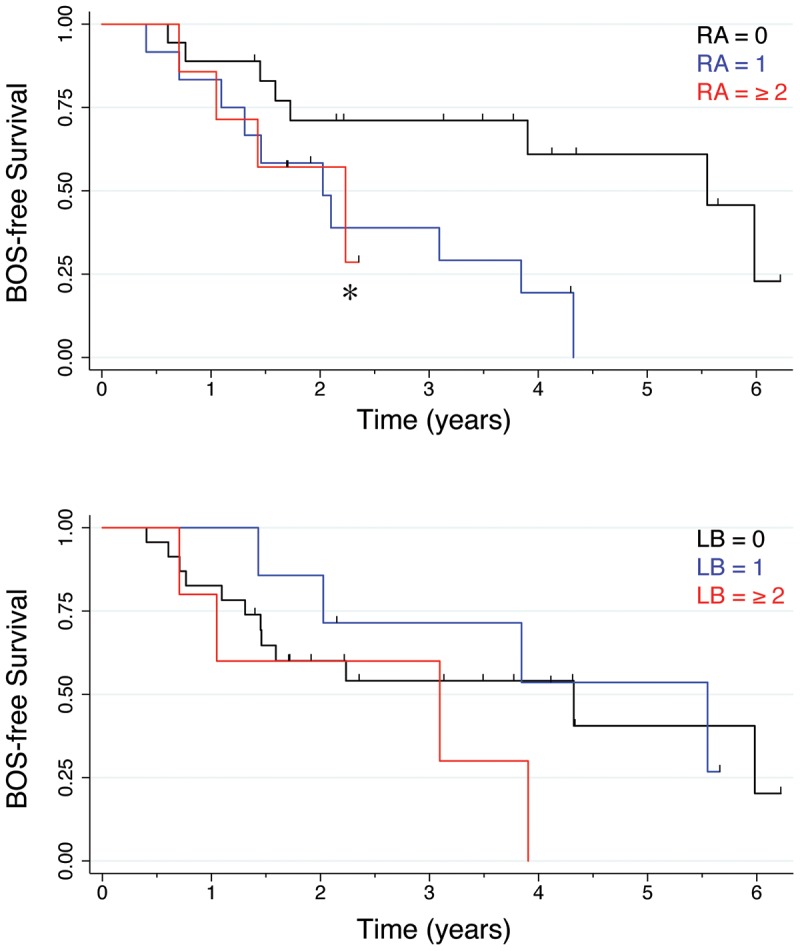
Kaplan-Meier analysis of BOS-free survival in 38 subjects based categorized by grade A rejection status (RA, top panel) or by grade of lymphocytic bronchiolitis (LB, bottom panel). *, P = 0.03 for RA by Mantel-Cox log rank test.

Both RA and LB were noted in a majority of subjects prior to the onset of BOS (Table 2). There was no difference in overall survival time based on either maximum grade RA or LB score in the first year after transplant. As the numbers of subjects with a RA grade of 2 or 3 were small, these were grouped in subsequent analysis. Three patients had a score of ≥2 for both RA and LB; two patients had a score of 1 for both RA and LB. The association between RA and LB was not significant as measured using Kendall's Tau test. There was a significant correlation between BOS-free survival time and maximum RA grade after transplantation (Figure 2) (P = 0.030 for RA by Mantel-Cox log rank test).

**Table 2:** Maximum grade A rejection (RA), lymphocytic bronchiolitis (LB), maximum HLA-G concentrations (U/ml), and presence of infection in 38 subjects prior to onset of BOS.

RA*, N (%)	
0	18 (48.6)
1	12 (32.4)
≥2	7 (18.9)
LB**, N (%)	
0	23 (65.7)
1	7 (20.0)
≥2	5 (14.3)
HLA-G, median (interquartile)	
Plasma	41.7 (10.6–74.0)
BAL	26.9 (6.8–49.2)
Number of infections, N (%)	
0	15 (39.5)
1	21 (55.3)
2	2 (5.3)

* 1 missing.

** 3 missing.

### sHLA-G concentrations and rejection

A total of 71 plasma and 85 BAL samples were collected in the first year after transplantation in 38 subjects. Plasma sHLA-G concentrations could be measured in every subject at every encounter, whereas 10 BAL samples had an sHLA-G concentration below the limit of sensitivity. Substantial variance was noted in both plasma and BAL maximum concentrations in the first year (Table 2).

**Figure 3:**
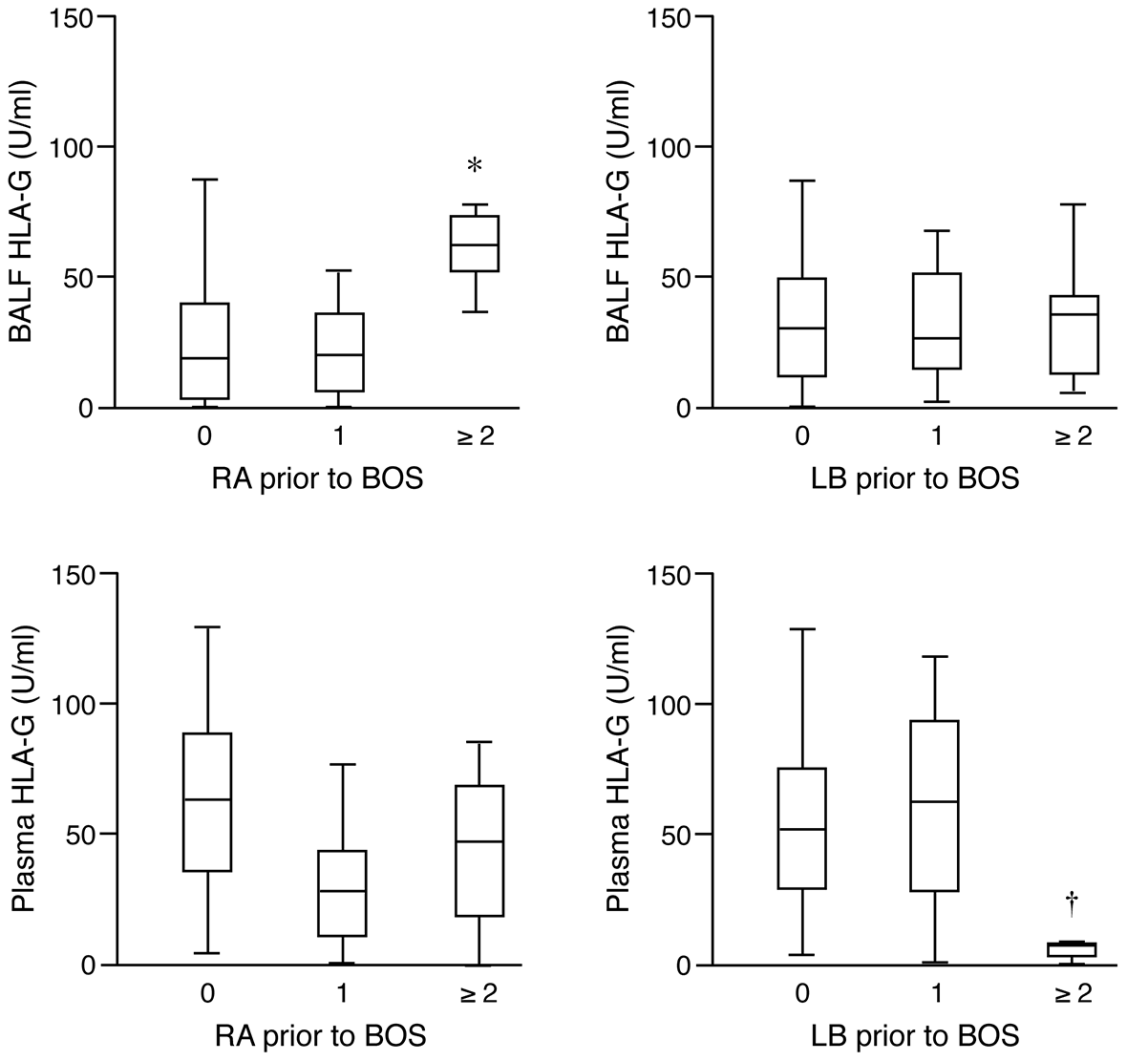
Concentrations of sHLA-G, in maximum U/ml prior to onset of BOS, in bronchoalveolar lavage fluid (BALF) (upper panels) and plasma (lower panels) in 38 subjects based categorized by grade A rejection status (RA, left panels) or by grade of lymphocytic bronchiolitis (LB, right panels). *, P = 0.006 by Kruskal-Wallis test, †, P = 0.044 by Kruskal-Wallis test.

There was no relation between either maximum plasma HLA-G concentration recorded in the first year, or in mean plasma HLA-G concentration of all first year samples, and overall survival or BOS-free survival. Likewise, there was no relation between either maximum BAL HLA-G concentration recorded in the first year, or in mean BAL HLA-G concentration of all first year samples, and overall survival. Contrary to our expectations, an increased maximum BAL HLA-G concentration was associated with a higher grade of RA prior to a clinical diagnosis of BOS (P = 0.006 by Kruskal-Wallis test) (Figure 3). In contrast, an increased maximum plasma HLA-G concentration was associated with a lower grade of LB prior to a clinical diagnosis of BOS (P = 0.044 by Kruskal-Wallis test), but not with any grade of RA (Figure 3).

### sHLA-G concentrations and infection

Both blood and lung infection, as demonstrated by positive cultures in blood or BAL fluid respectively, were noted in a majority of subjects prior to the onset of BOS (Table 2). There was no significant correlation between the number of infections and either plasma or BAL HLA-G concentrations.

## Discussion

Bronchiolitis obliterans syndrome remains the major limitation to long-term survival after lung transplantation despite advances in immunosuppressive therapy, infection control, and management of other complications. The poor prognosis associated with BOS reflects in part an inadequate understanding to date of disease processes which in turn leads either to under-treatment with immunosuppressive medications, and thus BOS progression, or to over-treatment or inappropriate treatment, and thus the increased number of infections and complications seen in this patient population. Our study demonstrates that the local (BAL) presence of HLA-G in the first year after transplantation in the lung correlates with the number of grade A rejection, and that circulating plasma HLA-G in the first year after transplantation correlates inversely with LB. Our study suggests that HLA-G may be a biological marker of rejection in a lung allograft. Such a marker, if confirmed in larger studies, would be useful to segregate those patients with a higher risk of rejection and BOS who require more intense immunosuppressive therapy from those in whom such therapy would entail increased risk without commensurate benefit.

Concentrations of sHLA-G were usually, but not always, detected in BAL fluid collected from LT recipients, and some variance was seen in BAL concentrations. We hypothesized that increased HLA-G levels in the lung would be associated with a lower rejection score as has been seen in patients following other solid-organ transplant [14]–[17]. Contrary to our hypothesis, however, the highest concentrations in BAL fluid were seen in patients with a RA score ≥2. The reasons for this are not clear: it may suggest that local production of HLA-G (by macrophages or by epithelial cells) is reactive and represent an attempt to induce the presence and generation of regulatory T cells [8]. Alternatively, it may reflect differences in the state of activation of airway macrophages and/or epithelial cells. Further evaluation of this will require studies in which local airway cell production can be ascertained over time.

There was variance also in circulating serum HLA-G concentrations in the first year after transplantation, and these were inversely associated with LB, but not RA, status. Serum HLA-G concentrations in subjects with a score of 1 in LB status was not different than that seen in recipients with a score of 0, while subjects with a score ≥2 had lower HLA-G concentrations. This suggests that mild lymphocytic bronchiolitis demonstrated on transbronchial biopsies may not be associated with changes in immune status sufficient to in turn decrease serum levels. The downward trend in serum HLA-G concentrations in patients with significant RA noted on transbronchial biopsy is not statistically significant but will need to be examined in the context of a larger study. Serum markers clearly are easier to obtain and, along with other serum markers, provide context for the overall status of the immune system. Correlations with circulating regulatory and effector T cell trafficking to the lung allograft will be needed to understand the potential role of serum sHLA-G in LT recipients.

In one recent single-center, retrospective study of 64 LT recipients within 1 year of transplant, HLA-G protein expression was seen in both bronchial and alveolar epithelial cells most frequently in stable patients but less so in patients with frequent AREs or in patients with BOS [19]. That study however did not evaluate the presence of either circulating sHLA-G in plasma or local sHLA-G in bronchoalveolar lavage. HLA-G also is found in lung macrophages [24], and evaluation of cell pellets by immunofluorescent staining collected from several LT recipients in our study demonstrated significant macrophage expression of sHLA-G (data not shown). Thus, sHLA-G found in bronchoalveolar lavage may represent the combined expression and secretion from both central and alveolar epithelial cells and from macrophages; changes in expression in either cell type might account for changes in the final presence in the lung. Understanding which cells contribute to final expression and presence after lung transplantation, and how that expression is modified by immunosuppressive drug treatment, infection and evolving chronic rejection, will require further study.

HLA-G induces expansion of CD4+FoxP3+ Treg cells [8] which may be important in allograft survival in transplantation. We have previously demonstrated that low numbers of lung FoxP3+ Treg cells are associated with accelerated rejection and the development of BOS in LT recipients [20]. Other investigators have demonstrated similar findings in stem cell [25] and bone marrow [26] transplantation, while increasing their number and function is associated with less alloreactivity in graft versus host disease [21]. Our new data raises the possibility that higher expression of *HLA-G* in the allograft may stimulate the presence and/or survival of lung Treg lymphocytes that then may modulate tolerance; this will need to be confirmed in future studies. In particular, our data do not make clear whether the change in allograft HLA-G expression is reactive, due to some rejection episode or stimulus from cells that ordinarily mediate rejection, or is innate and dependent more on a subject's (or allograft) genotype. HLA-G also inhibits both NK cell- and CD8+ T cell-mediated cytolysis [9], suppresses CD4+ T cell alloproliferative responses [10], and induces apoptosis of CD8+ T cells [11]. Likewise, HLA-G-bearing antigen-presenting cells not only inhibit CD4+ T cell proliferation but also induce the differentiation of CD4+ T cells into suppressor cells [12], [13]. Each action may improve allograft outcome, and will require evaluation in the context of LT.

Our study was too small to detect whether either plasma or local lung (BAL) HLA-G concentrations were associated with survival or BOS-free survival. Similarly, given the few time points that were collected for each subject and the missing samples, we were unable to assess changes in sHLA-G concentrations in BAL and serum reliably over time. Both limitations are typical of single-center studies and suggests that a multi-center trial with a significantly larger number of study subjects will be required to answer this question. Nevertheless our study does suggest that HLA-G is protective against LB; we predict that over a longer period of time and in larger studies that this will translate into improved BOS-free survival.

An additional issue is whether sHLA-G concentrations in BAL fluid reflect the local concentrations of sHLA-G at the tissue level. Previous studies in heart and lung transplant recipients have evaluated sHLA-G presence in endomyocardial and transbronchial biopsies, respectively [14], [19]. In the peripheral lung, sHLA-G presence in BAL fluid may reflect the combined relative contributions of airway epithelial cells [19] and of alveolar macrophages [24]. We did not directly compare the BAL concentrations to tissue presence of sHLA-G in this study, and both the correlation and the assignment of the BAL contribution to epithelial cell and/or macrophage origin will need to be addressed in future studies.

Our study also was too small to examine the potential relation of genotype on lung allograft function and the development of BOS. The influence of *HLA-G* genotype of both the donor graft and recipient has been examined in small studies of other solid organ transplants. While alleles encoding polymorphisms in the coding region apparently have little effect in renal transplantation [27], the 14 bp in/del polymorphism in exon 8 in the 3′ un-translated region of *HLA-G* may help predict renal [28] and bone marrow transplant [29] complications. Understanding how both donor and recipient genotypes influence tolerance will have important implications in matching donors to recipients in the future: as one example, if both genotypes predict low HLA-G expression, then clinicians may need to increase immunosuppressive therapy in terms of dosing or combinations of medications; and likewise, if either or both genotypes predict high expression, lower immunosuppressive therapy may be sufficient. Multi-center studies of *HLA-G* and its expression in lung allografts and in recipients will be needed to answer this question.

As may often happen in clinical studies with the collection of biological specimens, data were not available at each pre-specified time point because of the clinical condition (e.g., clinical instability during the bronchoscopy procedure) of the patient or logistics of specimen collection. However, the data remain useful despite these missing samples. In addition, we cannot exclude that different patient phenotypes or the variety of clinical events such as infection may bias our results. The putative use of lung sHLA-G as a biomarker for early allograft rejection clearly will require further study and confirmation in larger cohorts from different transplant centers.

In summary, we demonstrate the presence of sHLA-G in both the lung and serum of LT recipients. There is an association of high BAL sHLA-G presence and the development of grade A rejection within 2 years. While the mechanisms by which lung sHLA-G presence increases are not clear, this adaptive immunity mediator may be a marker of rejection in lung transplantation.
